# Double Interferometer Design for Independent Wavefront Manipulation in Spectral Domain Optical Coherence Tomography

**DOI:** 10.1038/s41598-019-50996-2

**Published:** 2019-10-10

**Authors:** Jonas Kanngiesser, Maik Rahlves, Bernhard Roth

**Affiliations:** 1 Leibniz Universität Hannover, Hannoversches Zentrum für Optische Technologien, Nienburger Straße 17, Hannover, D-30167 Germany; 2 Cluster of Excellence PhoenixD (Photonics, Optics, and Engineering - Innovation Across Disciplines), Hannover, D-30167 Germany

**Keywords:** Imaging techniques, Microscopy, Imaging and sensing, Adaptive optics

## Abstract

Spectral domain optical coherence tomography (SD-OCT) is a highly versatile method which allows for three dimensional optical imaging in scattering media. A number of recent publications demonstrated the technique to benefit from structured illumination and beam shaping approaches, e.g. to enhance the signal-to-noise ratio or the penetration depth with samples such as biological tissue. We present a compact and easy to implement design for independent wavefront manipulation and beam shaping at the reference and sample arm of the interferometric OCT device. The design requires a single spatial light modulator and can be integrated to existing free space SD-OCT systems by modifying the source arm only. We provide analytical and numerical discussion of the presented design as well as experimental data confirming the theoretical analysis. The system is highly versatile and lends itself for applications where independent phase or wavefront control is required. We demonstrate the system to be used for wavefront sensorless adaptive optics as well as for iterative optical wavefront shaping for OCT signal enhancement in strongly scattering media.

## Introduction

Optical coherence tomography (OCT) is a non-invasive optical technique for three-dimensional imaging with microscopic resolution. The method is based on the detection of light back-scattered from a sample. Utilizing a low coherence light source, such as a superluminescent diode (SLD), combined with interferometric signal acquisition allows to determine the optical path length of the reflected beam and, thus, to measure the distance between the scattering structure and the imaging system^1^. Spectral domain OCT (SD-OCT) enables simultaneous detection of photons reflected from different depths which yields a full axial line scan at the sample in case of point illumination. Lateral scanning of the illuminating beam allows for cross-sectional and volumetric mapping of the sample topography. Due to the capability for non-invasive three dimensional tissue imaging OCT has become a significant technique for medical diagnostics^2^, e.g. in the field of ophthalmology where the method acquires structural images of the clear parts of the eye as well as of the human retina^3–5^.

In case of transparent media present in front of the sample aberrations may disturb the illuminating as well as the back-scattered beam and, hence, cause a degradation of lateral image resolution. To overcome this limitation, adaptive optics has proven to be a powerful tool for OCT imaging^6–8^. The technique utilizes a wavefront shaping device such as a deformable mirror or a liquid crystal spatial light modulator (SLM) for wavefront manipulation at the sample beam to effectively correct optical aberrations. In case of retinal imaging the technique successfully compensates aberrations introduced by the anterior eye and, thus, creates a fine focal spot for high-resolution tissue imaging^6–8^. Typical approaches are based on the direct acquisition and compensation of the deformed wavefront returned from the sample^7,8^ or on sensorless wavefront optimization through acquisition of some image metric followed by wavefront optimization to enhance that metric^9^. Due to the finite lateral resolution of wavefront sensors and wavefront shaping devices typically employed, adaptive optics approaches are limited to the correction of low order aberrations and usually fail if it comes to imaging in strongly scattering inhomogeneous media, though.

The OCT imaging contrast results from detection of single-scattered photons. When imaging turbid media such as biological tissue the fraction of detectable single scattered light returned from the sample decreases with penetration depth^10^, resulting in the achievable imaging depth to be limited to the range of a few millimetres at most within biological tissue. Despite the highly random structure of such samples scattering is a deterministic process and manipulation of the optical wavefront incident to the sample yields control over the forward and backscattered field^11,12^. Recent publications demonstrated wavefront optimization techniques to iteratively shape the beam incident to the sample such that the OCT signal received from within strongly scattering media is enhanced^13–18^. In contrast to adaptive optics wavefront shaping does not require full correction of the highly scrambled wavefront transmitted through the sample but rather tries to control the phase of multiple independent wavefront segments such that constructive interference is created from the scattered beamlets^12,19,20^. The effect of the technique, thus, depends on the number of controlled wavefront segments, as does the run time of the iterative algorithm^12,19,21–26^.

Wavefront control further allows to create a set of orthogonal optical modes for sample illumination. Acquisition of the field scattered from the individual modes gives access to the sample’s complex-valued scattering matrix which describes the dependence of the scattered on the incident field^27^. The utilization of the scattering matrix for imaging of obstructed objects^28^ and for focusing behind scattering media by means of optical phase conjugation^27,28^ was demonstrated in previous works. Recent publications reported on the combination with coherence gating to acquire the depth-resolved reflection matrix in a way similar to spectral domain OCT^29^ as well as based on full-field OCT signal acquisition^30,31^. Based on the reflection matrix, the discrimination of multiple-scattered OCT signal components could be demonstrated, resulting in a clear advantage in signal-to-noise ratio (SNR) and achievable penetration depth over conventional signal acquisition with strongly scattering samples^30,31^.

All these techniques require wavefront control at the OCT sample and, in some cases, at the reference arm. We demonstrate an easy to implement design which allows for independent phase and wavefront modulation at both beams. The system is compact and only requires introduction of a wavefront shaping device at the source arm of a conventional free space SD-OCT system. Independent phase and wavefront control at the interferometer arms enable differential SD-OCT acquisition as well as a large number of OCT imaging applications such as beam steering, structured or multi-beam illumination, (sensorless) adaptive optics, iterative wavefront shaping, acquisition of the time-gated reflection matrix, optical phase conjugation as well as any combination of these techniques.

This paper is structured as follows: We first present a double interferometer design for independent wavefront manipulation at either arm of the OCT device. We follow up with an analytical and numerical discussion of the signal expected for this kind of system and provide experimental data confirming the theoretical considerations. Finally, we demonstrate applications of the system, including wavefront sensorless adaptive optics as well as iterative optical wavefront shaping.

## Double Interferometer OCT Design

**Figure 1:**
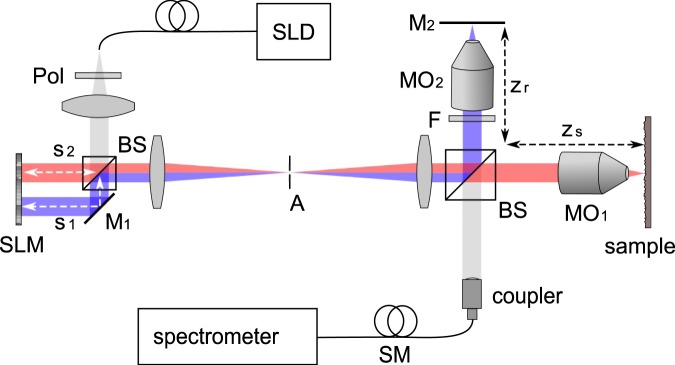
Proposed design. Abbreviations: *SLD* superluminescent diode, *Pol* linear polarizer, *BS* non-polarizing 50:50 beamsplitter, *SLM* spatial light modulator, *M* mirror, *A* aperture, *F* neutral density filter, *MO* microscope objective, *SM* single-mode optical fiber.

We illustrate the proposed free space SD-OCT design in Fig. 1. Light from a superluminescent diode (SLD) is linearly polarized and split by a 50:50 beam splitting cube. A spatial light modulator (SLM) reflects both beams at either half of its active area and, hence, allows to manipulate the beams independently. The beams are recombined and fed to a Linnik-type interferometer which features identical optical elements at both arms and, thus, effectively matches dispersion^32,33^. The fields reflected from the sample and the reference arm are coupled to a single-mode optical fiber and analysed by a spectrograph. The SD-OCT signal is recovered from the inverse Fourier transform of the acquired spectrum.

The first beam splitter together with the spatial light modulator forms an additional Michelson interferometer which affects the field incident to the Linnik interferometer. Such dual-interferometer OCT designs have been demonstrated for common-path OCT systems and are typically used to shift the axial field of view^34–40^. We demonstrate the design to enable independent wavefront control at the OCT system.

We consider *s*_1,2_ to be the arm lengths at the Michelson-type source interferometer and ${z}_{r,s}$ to be the arm lengths at the Linnik-type interferometer, respectively. The beam reflected from the bottom-half of the SLM and from the reference mirror M_2_ is delayed by $({s}_{1}+{z}_{r})/c$ (Fig. 1). Accordingly, the beam reflected from the top-half of the SLM screen and the sample is delayed by $({s}_{2}+{z}_{s})/c$. By choosing ${s}_{1} > {s}_{2}$ and ${z}_{r} < {z}_{s}$ we match the arm lengths such that ${s}_{1}+{z}_{r}={s}_{2}+{z}_{s}$. In this case the OCT system detects mutual interference from these two beams which we consider to be the effective sample and reference beam, respectively^35–40^. Manipulating the wavefront at either half of the SLM screen yields independent control of both beams. In case the arm length detuning $\Delta s={s}_{1}-{s}_{2}$ is chosen sufficiently large as compared to the axial field of view of the SD-OCT system, which is determined by the spectral resolution, additional signal components arising from the double interferometer design are suppressed^37,40^.

In principle, the design can be used with any type of spatial light manipulator such as a deformable mirror (DM) or a digital mirror device (DMD). We chose to use a liquid crystal on silicon (LCOS) SLM designed for phase only modulation. The device features a high spatial resolution and enables the application of multi-level phase patterns allowing for a variety of applications. In contrast to DMs and DMDs, LCOS devices typically suffer from a rather low response time and refresh rate, which often is limited to the range of a few Hz. For OCT signal acquisition the limited diffraction efficiency of the device due to reflection at the front surface of the liquid crystal layer and due to the limited fill factor as well as an eventual wavelength dependency of the applied phase shift have to be considered as well. The limited diffraction efficiency results in a fraction of the beam not being modulated. With our design this can be accounted for by slightly tilting the SLM and superimposing a linear phase ramp to the phase patterns applied at the SLM which compensates the tilt^41^. An aperture placed at the Fourier transform plane blocks the undiffracted beam and transmits the diffracted field only (Fig. 1). We experimentally investigated the wavelength dependency of the phase delay applied by the SLM and found it to be negligible at the spectral range ($820\mbox{--}860\,\mathrm{nm}$) covered by the SD-OCT system. Details on the SLM calibration and on the dispersion are given in the Supplementary Material. In case broadband sources such as femotsecond lasers or multiplexed SLD sources are to be used, the impact of the SLM dispersion has to be further investigated, however.

## Theoretical Analysis

Previous works provided detailed analysis of the double interferometer OCT design for common path systems^36–39^. The design was shown to effectively mimic a conventional single interferometer system in case that the optical path length difference at one interferometer compensates the path length difference at the other one^35–40^. We provide a short discussion to investigate the effect of the spatial light modulator on the system and to directly compare the double interferometer OCT signal with the signal expected for the conventional single interferometer device.

Spectral domain OCT signal acquisition can be described within the model of a one dimensional Michelson interferometer when the effect of the imaging optics is neglegted^1,42^. We consider the proposed design to be composed of a conventional SD-OCT system, which is the Linnik-type interferometer with arm lengths *z*_*r*_ and *z*_*s*_, and the additional Michelson interferometer with arm lengths *s*_1_ and *s*_2_. We assume the OCT signal expected for the conventional system, i.e. for the Linnik interferometer alone, to be ${I^{\prime} }_{d}(z)$. An extensive discussion on this signal is found in the standard literature^1^. The additional interferometer splits the source beam fed to the conventional SD-OCT system and applies a variable temporal delay depending on the arm lengths *s*_1_ and *s*_2_ to the beams, respectively. The spatial light modulator introduces a variable phase pattern ${\varphi }_{1,2}(x,y)$ to either beam upon reflection. Within the scope of the one dimensional model the phase delays ${\varphi }_{1,2}$ applied by the SLM are assumed to be uniform. In this case the A-scan signal expected for the double interferometer design is found to be

1 $${I}_{d}(z)=\frac{1}{4}[2R\,{{I}^{{\rm{^{\prime} }}}}_{d}(z)+R{e}^{i\Delta \varphi }\,{{I}^{{\rm{^{\prime} }}}}_{d}(z+2\Delta s)+R{e}^{-i\Delta \varphi }\,{{I}^{{\rm{^{\prime} }}}}_{d}(z-2\Delta s)]$$

where *R* is the intensity reflectivity of either source interferometer arm, $\Delta s={s}_{1}-{s}_{2}$ is the optical path length difference and $\Delta \varphi ={\varphi }_{1}-{\varphi }_{2}$.

As is evident from Eq. 1, the introduction of the additional interferometer causes three distinct copies of ${I^{\prime} }_{d}(z)$ to be detected at different depths *z* in the A-scan. The first term corresponds to an incoherent superposition of the fields reflected from both arms of the source interferometer. The conventional A-scan signal ${I^{\prime} }_{d}(z)$ is reproduced at its original z-scale and the signal intensity is scaled by the interferometer’s total incoherent intensity reflectivity $2R$. In case either arm of the source interferometer is blocked, this signal component is the only one to persist. The second and third terms correspond to mutual interference of the two beams reflected at the source interferometer. The corresponding A-scan signal components are, thus, sensitive to the arm length detuning $\Delta s$, which determines the signal shift along the z-axis, as well as to the phase patterns applied to the respective beams by the SLM. In case of uniform phase delays ${\varphi }_{1,2}$ the OCT signal is sensitive to the phase difference $\Delta \varphi ={\varphi }_{1}-{\varphi }_{2}$.

We illustrate the impact of Eq. 1 with semi-analytic simulations of the expected OCT signal. We calculated the spectral power density at the detector of the double-interferometer design based on the one dimensional model of the system and for a sample featuring multiple reflective layers. The complex-valued A-scan signal is retrieved by taking the inverse Fast Fourier Transform (IFFT) of the real-valued spectrum. The complex OCT signal, thus, is expected to be hermitian symmetric. The finite resolution of the spectrograph is neglegted. To clearly identify the individual signal components evident in Eq. 1 we repeated the calculation for different phase shifts $\Delta \varphi$ introduced by the SLM and calculated the resulting phase shift of the recovered complex-valued OCT signal. Details on the simulation are given in the Supplementary Material.

**Figure 2:**
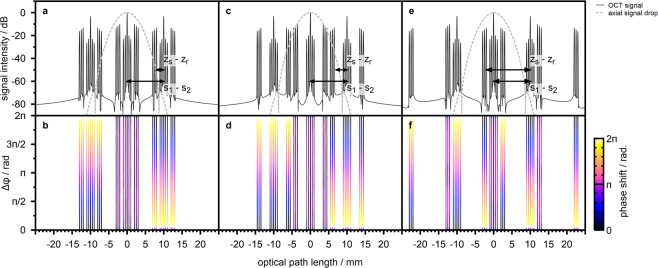
Semi-analytic calculation of the expected SD-OCT signal for a sample of three reflective layers and an arm length detuning $\Delta s=10\,{\rm{mm}}$ at the source interferometer. (**a**) OCT-signal in case the reference and sample arm lengths ${z}_{r}$ and ${z}_{s}$ approximately match. The effect of finite spectral resolution is neglegted. The dashed line indicates the axial signal drop due to the finite resolution of the spectrograph which is modelled by the product of a Gaussian and a *sinc* function. (**b**) Phase of the A-scan signal in case the phase difference $\Delta \varphi$ at the source interferometer is changed. The data is thresholded to signal components with amplitude larger than −60 dB. (**c** and **d**) A-scan amplitude and phase in case the reference arm length ${z}_{r}$ is shortened by 1.5 mm. (**e** and **f**) A-scan amplitude and phase in case the reference arm is shortened by 10 mm, matching the arm length detuning Δ*s* at the source interferometer.

Figure 2a illustrates the A-scan signal amplitude expected for a source interferometer arm length detuning Δ*s* = 10 mm, which is close to the experimental design, and for a sample of three reflective layers with layer spacing 0.5 mm and decreasing reflectivity. According to conventional OCT data processing we rescaled the optical path length axis by a factor 0.5 as compared to the theoretical discussion (Eq. 1). We find the peak signal-to-noise ratio of the simulated OCT signal to be determined by the spectral range considered for the calculation which we chose to be four times the source FWHM bandwidth. Figure 2b additionally illustrates the dependence of the signal phase on Δ$\varphi$.

The OCT signal observed near $z=0$ reflects the signal ${I^{\prime} }_{d}(z)$ expected for a conventional SD-OCT system. This signal results from incoherent superposition of the two beams reflected at the source interferometer and, thus, does not depend on the phase shift Δ$\varphi$ introduced by the SLM. A strong DC peak and autocorrrelation signal components close to the DC signal are evident. Signal components resulting from mutual interference of the reference arm ${z}_{r}$ and the sample arm ${z}_{s}$ are observed at path lengths between 2 mm and 3 mm and feature a higher amplitude as compared to the autocorrelation signal.

The additional Michelson interferometer causes copies of the single-interferometer SD-OCT signal ${I^{\prime} }_{d}(z)$ to be detected at shifted depth positions. These signal components correspond to mutual interference of the two beams reflected at the source interferometer, i.e. at either half of the SLM screen, and are sensitive to the phase shift Δ$\varphi$ introduced at the SLM. The axial displacement in the OCT signal matches the arm length detuning Δ*s*. The mutual interference components of the shifted signal is of special interest. These signal components correspond to mutual interference of the beam reflected at one half of the SLM and at the reference arm *z*_*r*_ and the beam which is reflected at the other half of the SLM screen and at the sample. Selective detection of these signal components, thus, allows for OCT signal acquistion with independent wavefront manipulation at the reference and at the sample beam, respectively.

As is evident from Fig. 2, the double interferometer design causes the expected SD-OCT signal to be spread over a large depth range. Practical implementations of SD-OCT systems suffer from a limited axial field of view, however. In principle the observable depth range ±*z*_*max*_ is determined by the spectral sampling interval of the spectrograph^1,42,43^. The discrete Fourier transform which is used to calculate the A-scan signal from the acquired spectrum causes the OCT signal to be periodic in that range, i.e. ${I}_{d}(z)={I}_{d}(z+2n\,{z}_{max})$ for integer numbers *n*. As a consequence, signal components beyond ±*z*_*max*_ appear to be wrapped to the observable axial imaging range. Considering Fig. 2, this results in all signal components to be detectable with the double interferometer SD-OCT system in principle.

Besides the spectral sampling interval, practical systems feature a finite spectral resolution due to the spectrometer optics and due to the pixel size of the detector which is imaging the spectrum. The finite resolution suppresses fine spectral features which correspond to OCT signals at large optical path length differences and, thus, acts as a low-pass filter on the acquired A-scan^1,42^. The effect of finite pixel size can be modelled by a *sinc* function centered at $z=0$ which describes the axial signal drop^42^. Correspondingly, the effect of finite optical resolution is modelled by a Gaussian function^1^. Figure 2 illustrates the axial signal drop expected in case the parameters of the spectrograph used for the experimental device are taken. We assumed the FWHM resolution of the spectrometer optics to be $\delta {k}_{opt}=6.58\,{{\rm{cm}}}^{-1}$ and the spectral range covered by the individual detector pixels to be $\delta {k}_{pix}=3.61\,{{\rm{cm}}}^{-1}$. With these parameters, the OCT signal is expected to be suppressed by more than −60 dB at path length differences beyond 8 mm.

The finite axial field of view of the SD-OCT system can be harnessed to mimic the behaviour of a conventional SD-OCT design^37,40^. In case the arm length detuning Δ*s* is chosen sufficiently large, DC and autocorrelation components of the shifted signals are moved beyond the axial imaging range of the system (Fig. 2). These signal components are suppressed due to the limited spectral resolution and, thus, do not affect the acquisition. The position of the mutual interference signal is determined by the path length difference ${z}_{s}-{z}_{r}$ between sample and reference arm, on the other hand. By changing the reference arm length the axial position of the mutual interference signal resulting from interference of the beam reflected at one half of the SLM and at the reference arm ${z}_{r}$ and the beam which is reflected at the other half of the SLM screen and at the sample can be moved within the axial imaging range of the SD-OCT system by choosing ${z}_{s}-{z}_{r}\approx {s}_{1}-{s}_{2}$ (Fig. 2e). In this case, the mutual interference signal detected by the SD-OCT system results from two beams which can both be independently shaped by the SLM and which are reflected at the sample and at the reference mirror, respectively. The DC and autocorrelation artefacts detected close to $z=0$ result from incoherent superposition of the beams reflected at the source interferometer (first term of Eq. 1). This signal is scaled by a factor 2 as compared to the detected mutual interference components which result from the second and third term in Eq. 1. The double-interferometer SD-OCT design, thus, allows independent beam shaping at the price of stronger imaging artefacts as compared to a conventional SD-OCT system^37,40^.

## Phase Sensitive OCT Signal Acquisition

**Figure 3:**
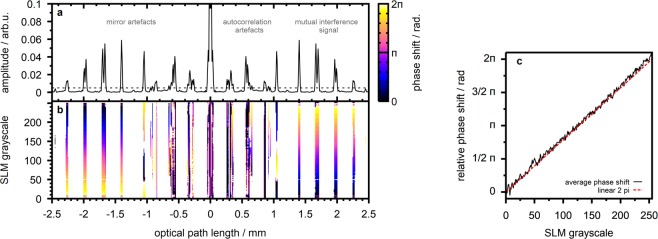
SLM-based phase shifting. (**a**) Observed OCT signal amplitude for a flat sample with four reflecting interfaces. (**b**) Observed OCT signal phase in case of wavefront manipulation at the bottom half of the SLM screen (reference arm). The phase is illustrated for those positions where the signal amplitude is larger than 0.5% of the DC peak amplitude. (**c**) Average phase shift at the mutual interference signal components, i.e. above the amplitude threshold and between $z=1.4$ and $z=2.3\,{\rm{mm}}$.

This Section is intended to experimentaly verify the previous calculations. We used the double interferometer OCT setup to image a layered sample consisting of three cover glass slides (CG15CH2, Thorlabs, USA) attached to an objective slide. Figure 3a illustrates the A-scan taken with the sample placed at the focal plane of objective lens MO_1_. The signal features four prominent peaks corresponding to reflections at the sample interfaces located at depths of 1.4, 1.7, 2.0 and 2.3 mm in the A-scan, respectively. Closer to the $z=0$ position a number of artefact signals are evident.

We utilized the phase only spatial light modulator to manipulate the phase difference Δ$\varphi$ between the source interferometer arms by applying a uniform grayscale image to the bottom half of the SLM screen (compare Fig. 1). This procedure is expected to correspond to phase shifting at the OCT reference arm, i.e. rising Δ$\varphi$ in case the grayscale value is increased. The resulting phase of the SD-OCT signal, which is calculated from the IFFT of the acquired real-valued spectrum, is illustrated in Fig. 3b. The figure clearly reveals the mutual interference signal components affected by phase shifting at the SLM whereas the phase at the artefact signals shows no systematic dependence. Figure 3c illustrates the average phase at the mutual interference signal peaks for the different grayscale values applied to the SLM screen. We observe the OCT signal phase to agree well with the linear 2*π* phase modulation characteristics for which the SLM was calibrated. We, thus, conclude that the SLM allows to accurately manipulate the phase of the observed OCT signal.

## Differential OCT

Since the mutual interference OCT signal component is the only one to be affected by the phase difference Δ$\varphi$ between reference and sample arm, phase shifting allows to suppress unwanted signal components such as DC, autocorrelation and mirror artefacts and to increase the signal-to-noise ratio^44–50^. The technique can, thus, be used to compensate for increased artefact intensities with the double interferometer design. Typical approaches vary in the type of phase modulation as well as in the number of phase shifted signals to be used for image reconstruction^44–52^. In our previous work we demonstrated the implementation of a four step algorithm^1^ with a setup similar to the presented design^18^. In principle, the phase only SLM can be used to introduce an arbitrary phase shift, however (Fig. 3).

In contrast to comparable systems for phase sensitive OCT signal acquisition, our approach is affected by the free space design resulting in phase fluctuations as well as by the slow response time of the liquid crystal SLM which is in the range of 100 ms and significantly limits the achievable imaging speed. The artefact suppression ratio of the algorithms depends strongly on the accuracy of phase shifting and on the phase stability during acquisition. Full artefact suppression, hence, is not observed with the current implementation of our experimental system^18^. On the other hand, the experimental design allows to superimpose the uniform phase shift required by the technique with any other arbitrary phase pattern applied by the SLM at the same time^18^. The approach, thus, enables the combination of beam shaping techniques with differential SD-OCT acquisition without the need for additional components.

## Wavefront Sensorless Adaptive Optics

We demonstrate the beam shaping capability of our setup with an algorithm for sensorless adaptive optics. We implemented an iterative feedback-based algorithm similar to the one presented by Jian *et al*.^9^. The algorithm calculates a feedback signal from the integrated OCT signal received from an arbitrarily chosen depth window. In contrast to the previous work, the algorithm is based on a single A-scan signal as opposed to the implementation based on a volume scan^9^ due to the slow scanner employed in our system. The algorithm sequentially optimizes the Zernike coefficients from which the corrective wavefront is composed individually such that the feedback signal is maximized. For the proof-of-concept experiment presented we implemented correction up to the 4th radial order applied either to the reference or to the sample beam. The feedback signal can be calculated from the intensity of the conventional OCT scan or from the differential signal based on phase shifting at either beam.

**Figure 4:**
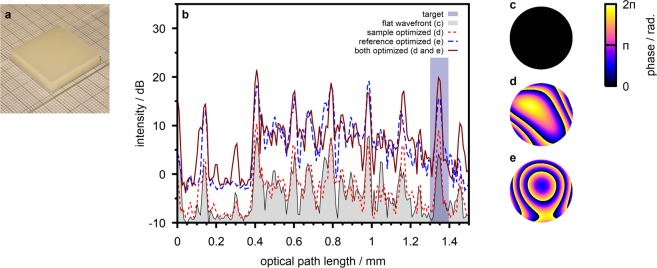
Wavefront sensorless adaptive optics. (**a**) Sample photograph. (**b**) A-scans taken at the sample with different phase patterns applied. The depth-range from which the feedback signal for wavefront optimization is calculated is highlighted. (**c**) Flat wavefront applied to both beams for the acquisition of the initial scan. The pattern corresponds to a beam diameter of 4 mm at the SLM. (**d**) Wavefront optimized at the sample beam. (**e**) Wavefront optimized at the reference beam.

We demonstrate the technique with a turbid sample prepared from multiple layers of scattering polymer film (Parafilm M, Pechiney Plastic Packaging, USA) sandwiched between a cover glass and an objective slide (Fig. 4a). We found the sample extinction coefficient to be $(6,9\pm 1,2)\,{{\rm{mm}}}^{-1}$ from forward scattered transmission experiments. We further, on purpose, introduced static aberrations to the reference beam by slightly displacing the objective lens MO_2_ from the reference mirror M_2_ (compare Fig. 1). The aberrations result in the detected reference arm power to be lower than the power returned from the sample.

Figure 4b illustrates the OCT signal taken at the sample with a flat wavefront (Fig. 4c) applied to both beams. To increase the signal-to-noise ratio, which is very low with the conventional SD-OCT signal due to the defocus at the reference arm and the resulting loss of reference power, the scan was taken with a four step phase shifting algorithm (compare Sec. 4). We utilized the adaptive optics algorithm to optimize the differential OCT signal corresponding to the interface between the fifth and the sixth scattering polymer layer which is observed at a depth of 1.35 mm in the A-scan. Figure 4b illustrates the signal received in case the wavefront at the sample beam is optimized (Fig. 4d). We observe a peak intensity enhancement of 3.8 dB at the target position. We repeated the experiment with wavefront correction at the reference beam which yields a peak intensity enhancement of 11.7 dB at the target depth. The optimized wavefront (Fig. 4e) reflects the aberrations at the reference beam which are expected to be dominated by defocus. We finally acquired the OCT signal with both optimized phase patterns applied to the respective beams at the same time. The OCT signal features a signal enhancement of 15.0 dB in this case.

## Iterative Wavefront Shaping

To better illustrate the impact of wavefront correction at the sample and reference beam we further implemented an iterative wavefront shaping algorithm. Iterative wavefront shaping was developed for focusing and imaging in strongly scattering media^19^. The technique does not try to fully correct for aberrations introduced by the sample but rather controls the phase of individual wavefront segments incident to the medium. Due to the deterministic propagation of light, constructive interference can be created from the scattered wavelets to shape a focal spot inside or behind the highly heterogeneous sample^12,19,20^. In contrast to adaptive optics, the technique requires single-pass wavefront control and the efficiency depends on the number of controlled independent wavefront segments^12,19,21–26^.

Recent work demonstrated the combination of iterative wavefront shaping with optical coherence tomography^13–18^. The technique was shown to be able to locally enhance the OCT signal acquired from a strongly scattering medium and, thus, to increase the penetration depth in turbid samples. We implemented OCT-based iterative wavefront shaping relying on the genetic algorithm presented by Conkey *et al*.^25^. As with the adaptive optics algorithm presented previously, we calculate a feedback signal from the integrated A-scan intensity at an arbitrarily chosen target depth window. The algorithm groups the phase pattern at the SLM to a number of independent wavefront segments and applies an iterative genetic algorithm to optimize the phase of the individual segments such that the received feedback, i.e. the OCT signal at the target, is maximized^18,25^.

**Figure 5:**
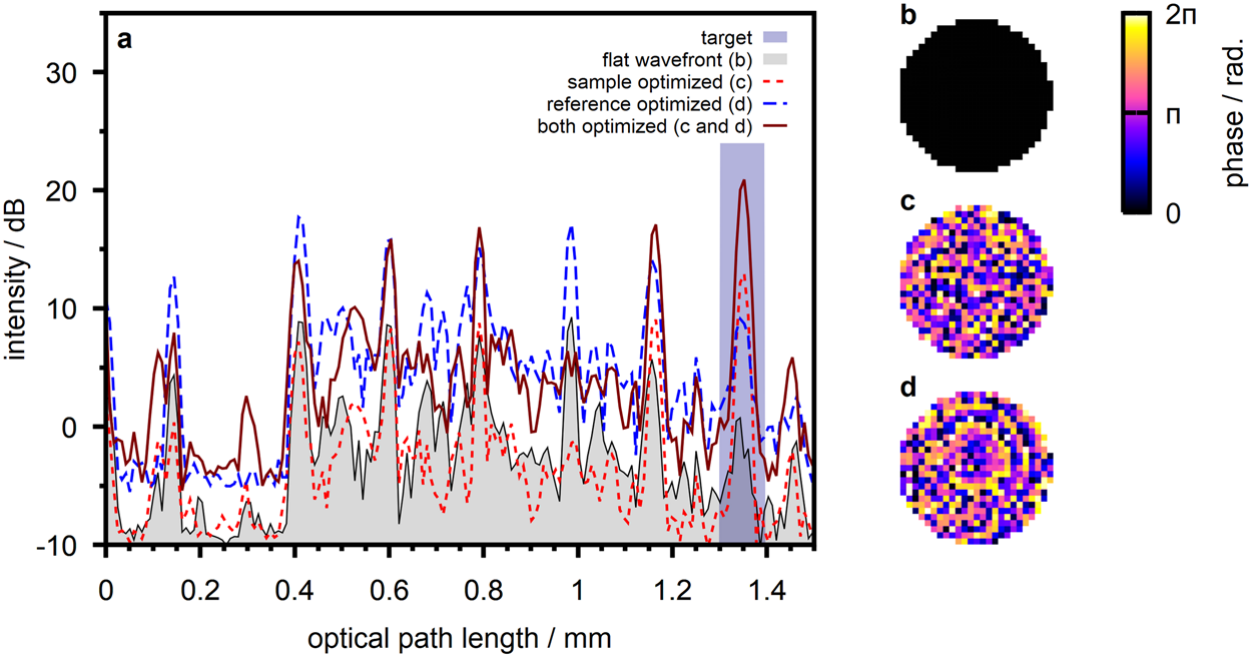
Iterative wavefront shaping. (**a**) A-scans taken at the sample with different wavefront applied to the reference and sample beams. (**b**) Flat wavefront applied to both beams for the acquisition of the initial scan. (**c**) Wavefront optimized at the sample beam. (**d**) Wavefront optimized at the reference beam.

We demonstrate the technique with the previous setup, i.e. with a layered scattering sample and a defocused reference beam. We calculated the feedback for the wavefront shaping algorithm from the differential OCT signal based on four step phase shifting. Figure 5 illustrates the resulting OCT signal in case the wavefront is optimized at the sample and at the reference beam, respectively. For the data presented we optimized a number of 493 independent wavefront segments. With optimization at the sample beam the algorithm yields a 10.0 dB increase of the target signal. Wavefront shaping at the reference beam yields a 8.4 dB increase. In case both optimized phase patterns are applied at the respective beams simultaneously, a 18.0 dB signal increase is observed.

**Figure 6:**
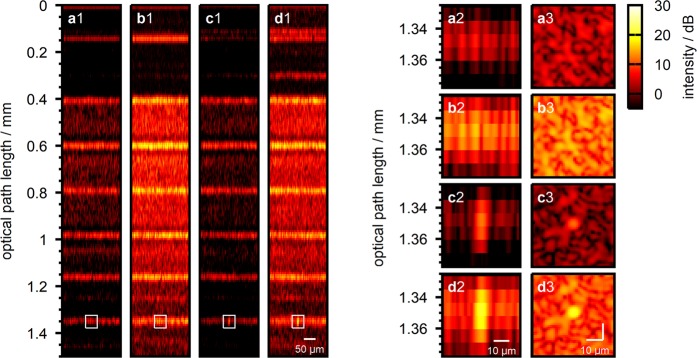
Lateral sample scan with shaped wavefronts applied. (**a1**) B-scan taken with flat wavefront applied to both beams. (**a2**) B-scan close-up centered at the target scan position which was chosen for wavefront optimization. (**a3**) En-Face scan at the target depth ($z=1.35\,{\rm{mm}}$) for wavefront optimization. (**b1**–**b3**) Same as (**a1**–**a3**) with wavefront optimized at the reference beam (Fig. 5d). (**c1**–**c3**) Same as (**a1**–**a3**) with wavefront optimized at the sample beam (Fig. 5c). (**d1**–**d3**) Same as (**a1**–**a3**) with both wavefronts applied to the respective beams simultaneously.

The impact of the shaped wavefronts becomes apparent when the sample is scanned with the static wavefront applied. Figure 6, panels a1–a3 and panels b1–b3 illustrate the B-scan and an En-Face scan taken at the target depth with the flat wavefront (Fig. 5b) and with the wavefront optimized at the reference beam (Fig. 5d) applied. The B-scans clearly reveal the sample’s layered structure. We observe the total OCT signal intensity to be enhanced with the optimized wavefront applied to the reference beam due to the increased reference beam power coupled to the spectrograph. The imaged structure essentially stays the same, on the other hand. Indeed, we find the image correlation of the two En-Face scans (Fig. 6, panels a3 and b3) to be 0.87. In contrast, in case of wavefront shaping at the sample beam (Fig. 5c) the signal is observed to be enhanced at the three-dimensional target position for which the wavefront was optimized only (Fig. 6, panels c1–c3). We find the En-Face image correlation with the scan taken with flat wavefront applied (Fig. 6, panels a3 and c3) to be −0.02, i.e. both images are uncorrelated. In case both optimized wavefronts (Fig. 5c,d) are applied to the respective beams simultaneously, we observe both effects to cumululate (Fig. 6 panels d1–d3). An overall signal intensity enhancement due to shaping of the reference beam as well as a localized signal enhancement due to wavefront shaping at the sample is observed. We find the correlation coefficient between the En-Face scans displayed in Fig. 6, panels c3 and d3 to be 0.96.

## Discussion

The sensorless adaptive optics algorithm is observed to effectively correct for the aberrations introduced to the reference beam which are expected to be dominated by defocus (Fig. 4e). The algorithm is based on the enhancement of the observed OCT signal intensity. In our experiments it allows to correct for aberrations at the reference beam because the detected reference beam power was lower than the sample beam power and a significant signal increase can be observed when the optimized wavefront is applied. In contrast, the adaptive algorithm yields only a minor increase in OCT signal intensity in case the wavefront at the sample beam is optimized. This is due to the low order of aberrations which are corrected by the algorithm with the current implementation. The algorithm, thus, finds a rather smooth wavefront (Fig. 4d) which does not sufficiently account for the inhomogeneous structure of the scattering sample.

The iterative wavefront shaping algorithm is designed for inhomogeneous scattering samples and random-like corrective wavefronts, on the other hand. In case of wavefront shaping at the reference beam, the algorithm finds a wavefront (Fig. 5d) similar to the adaptive algorithm (Fig. 4e). Due to the random nature of the iterative optimization algorithm some phase fluctuations arise and the phase pattern is not found to be smooth. The resulting signal enhancement is observed to be lower than with the adaptive algorithm (8.4 dB vs. 11.7 dB). In case of wavefront shaping at the sample beam the algorithm finds a highly heterogeneous phase pattern (Fig. 5c) which can not be obtained by the adaptive algorithm. The heterogeneous wavefront results in a significant signal increase at the target (10.0 dB) as opposed to the smooth pattern produced by the adaptive algorithm (3.8 dB).

Wavefront shaping at the reference beam results in an overall enhancement of the OCT signal which does not depend on the actual position of the scanning beam at the sample (Fig. 6 panels b1–b3). In contrast, the effect of iterative wavefront shaping at the sample beam is limited to the three dimensional target position for which the wavefront is optimized (Fig. 6 panels c1–c3). The observed signal enhancement is an effect of constructive interference of the wavefront which is scattered at the heterogeneous sample structure. When the beam is scanned the scattered field decorrelates as does the effect of the shaped wavefront^18,20^. This behaviour is not observed with the adaptive wavefront correction (compare Supplementary Fig. S4) and proves that the wavefront indeed is optimized to match the scattering sample structure.

With the proof-of-principle experiments presented, wavefront optimization took approximately 15 minutes in case of the adaptive algorithm and one hour in case of the iterative optimization algorithm. The long optimization time is due to the slow response time of the liquid crystal SLM and to the algorithms not being tuned for a low number of iterations with the current implementation. The combination with signal acquisition based on four-step phase shifting additionally increases the acquisition time.

## Conclusion

We demonstrated a technique for independent wavefront manipulation at the sample and reference arm of a spectral domain OCT device based on a single spatial light modulator. The optical design can easily be implemented to existing free space SD-OCT systems through introduction of an additional interferometer at the light source. We provided analytical and numerical discussion of the OCT signal expected with this setup and demonstrated experimental results to verify the analysis. The system allows for independent single-pass wavefront manipulation and beam shaping at either arm of the interferometer as well as, in case a phase-only spatial light modulator is used, for independent phase manipulation. The design, hence, is highly versatile and lends itself for a large number of different applications which can be digitally switched by changing the pattern applied to the SLM.

We demonstrated the system to be used for manipulation of the OCT signal phase by applying uniform phase patterns at the SLM. This allows for the suppression of OCT imaging artefacts and for enhancement of the SNR based on the sequential acquisition of multiple phase shifted spectra. This comes at the cost of increased acquisition times, however. For the experiments presented we implemented a four-step phase shifting algorithm. We further implemented a feedback based algorithm for the correction of low-order optical aberrations as well as a feedback based iterative wavefront shaping algorithm for signal enhancement with strongly scattering media. Both algorithms were implemented for correction at the sample and at the reference beam and allow the conventional OCT signal or the OCT signal received from phase shifting algorithms to be used for the calculation of the feedback signal. We demonstrated both algorithms to correct for static aberrations introduced to the reference beam. The iterative wavefront shaping algorithms was further shown to locally enhance the OCT signal received from a scattering sample. A major challenge to be considered is the long acquisition time with the current implementation of both wavefront shaping algorithms. For imaging applications this can be addressed by implementing improved algorithms^9,13–16^ and by utilizing faster spatial light modulators. We would further like to point out that the experimental design was developed for iterative wavefront shaping and, hence, enables single-pass wavefront manipulation as opposed to double-pass manipulation which is typically found with adaptive optical imaging systems. Adaptive wavefront correction algorithms, hence, may have to be further tuned to account for this kind of modulation.

Beyond the demonstrated applications, the design allows for beam shaping at the OCT system, e.g. by applying a Bessel beam to the sample^53,54^ or multiple parallel beamlets. Since the reference beam has to be superimposed with the reflected sample beam at the detector, the technique requires to adapt the reference beam to the illumination scheme. With the presented design, this can easily be achieved with the spatial light modulator and without the need for changing the optical design. The SLM enables the application of diffractive phase patterns and, hence, the implementation of arbitrary illumination schemes beyond the scope of conventional bulk optics as well as fast digital switching between them. The design is further suited for applications such as the acquisition of the depth-resolved reflection matrix, which describes the scattering properties of a sample and which can be used for OCT imaging with strongly scattering media^29–31^.

We believe that the techniques based on the acquisition of the reflection matrix^29–31^ or on iterative wavefront shaping^13–16^ can increase the OCT penetration depth and imaging performance in strongly scattering media. We are, thus, confident that the presented system will enable novel implementations and extensions of these techniques, for example the combination with parallelized multi-beam acquisition, and to allow for deep tissue OCT imaging.

## Methods

### Experimental setup

The OCT design is based on a Linnik-type interferometer, which is a Michelson interferometer with equal objective lenses at both interferometer arms (right part of Fig. 1a). Although this layout typically is found in full field OCT systems^1,32,33^ it features advantages for spectral domain OCT applications as well. Namely, it allows for alignment of the reference arm focal position independently from the reference arm length and introduces equal aberrations and dispersion to both interferometer arms. The system is fed by a broadband light source centered at 830 nm (SLD830S-A10, Thorlabs, USA). The light back-scattered from the sample is overlapped with the reference beam, coupled to a single-mode optical fiber and analysed by a spectrograph (SR500i with DV-420A-OE camera, Andor, United Kingdom) with an average nominal wavenumber sampling of $\Delta k=3.61\,{{\rm{cm}}}^{-1}$, resulting in a single-sided OCT imaging depth of 4.4 mm. Details on the spectral resolution are given in the Supplementary Material. For precise alignment and lateral scanning the sample is mounted to a 3-axis piezoelectric stage (P-611.2s and P-622.ZCD, Physik Instrumente, Germany).

To manipulate the optical wavefront at the SD-OCT system we include a phase-only spatial light modulator (HEO1080P with NIR11 display, Holoeye, Germany) which reflects both beams at an additional Michelson interferometer located at the OCT source beam (Fig. 1). The path length difference at the Michelson interferometer is chosen to be $(9.7\pm 0.1)\,{\rm{mm}}$ which is well beyond the axial imaging range as determined by the spectral resolution. The SLM is imaged to the objective lens MO_1_ pupil plane using a 4f system. Its screen is aligned to the orientation of the linearly polarized source and perpendicularly illuminated to assure performance close to its specifications. The SLM was calibrated for linear 2*π* phase modulation with the SLD source. Details on the calibration procedure are given in the Supplementary Material.

### Four-step algorithm for differential OCT signal acquistion

We implemented a four step algorithm taken from literature^1^ for differential signal acquisition. With that method four spectra ${i}_{d}(k,\Delta \varphi )$ are acquired with phase differences $\Delta \varphi$ applied between the reference and sample beam. From that data the complex valued spectral interferogram ${\tilde{i}}_{d}(k)$ is calculated: ${\tilde{i}}_{d}(k)={i}_{d}(k,0)-{i}_{d}(k,\pi )-i[{i}_{d}(k,\pi /2)-{i}_{d}(k,3\pi /2)]$. The corresponding A-scan is acquired from the inverse Fourier transform of that signal and suppresses DC, autocorrelation and mirror artefacts. We implemented phase shifting at the reference and at the sample beam, respectively. In case of phase shifting at the sample beam, the signal is calculated from the complex conjugate of the previous equation.

### Sensorless adaptive optics

We implemented adaptive wavefront correction similar to the method presented by Jian *et al*.^9^. The algorithm tries to find an optimized wavefront such that a scalar feedback signal is maximized. We calculate the feedback from the integrated A-scan signal received from a user-defined depth range. The phase pattern for wavefront correction is assumed to be a linear combination of Zernike modes. The algorithm starts with a flat phase pattern and optimizes the coefficient of each mode individually.

Starting with the first mode we assign a set of linearly spaced sample-coefficients. The corresponding phase patterns are found from the product of the respective sample coefficients and the Zernike mode under test. The algorithm applies the phase patterns to the SLM and acquires the resulting OCT signal. From that data, the sample coefficient resulting in the maximal feedback signal is found. The procedure is repeated for the same mode with a new set of sample coefficients centered at the previous best value and with a reduced sampling interval. Once the optimal coefficient for the mode under test is found, the corresponding phase pattern is applied to the corrective wavefront and the algorithm proceeds with the next mode. For subsequent modes, the phase pattern is superimposed to the preliminary optimized wavefront. The algorithm, thus, is sensitive to the sequence of modes to be optimized,

The algorithm allows user selection of the number of sample coefficients, the initial range of coefficients and the sequence of modes. For the data presented we chose a number of 11 sample coefficients equally spaced at the initial range between −5 and 5. The algorithm started with the correction of defocus, astigmatism, tip and tilt before optimizing the residual Zernike coefficients up to the 4th radial order.

The feedback signal taken for the algorithm is calculated from the conventional SD-OCT signal or from the differential signal. In the latter case, phase shifting can be implemented with wavefront manipulation at both beams, i.e. by optimizing the wavefront at one beam and and using the other beam for phase shifting, or with wavefront shaping at one beam only. This is achieved by superimposing a constant phase offset to the non-uniform phase pattern which is used for wavefront optimization.

### Iterative wavefront shaping

As with the adaptive wavefront optimization, the iterative wavefront shaping algorithm tries to find an optimized wavefront such that a feedback value, which is the integrated A-scan intensity at a user selected depth range, is maximized. The algorithm groups the wavefront to independent segments with uniform phase, respectively. The phase of the individual segments is found with an iterative genetic optimization procedure^18,25^. We start by creating a population of randomly chose phase patterns. The feedback values resulting from illumination with the respective wavefronts are acquired. The algorithm proceeds by creating generations of new phase patterns by randomly selecting two patterns from the previous generation and blending them. The selection probability is chosen to be higher for patterns resulting in a larger feedback signal. Additionally some random phase fluctuations are introduced to new patterns upon creation. As with the adaptive optimization, the feedback signal taken by the algorithm can be calculated either from the conventional SD-COT signal or from the differential signal.

For the data presented we optimized a wavefront consisting of 493 segments. We created 50 phase patterns for the initial population and ran the algorithm to create 150 generations with 20 new phase patterns, respectively.

## Supplementary information

Supplementary Information

## Data Availability

The simulation code generated during the current study is available in the Figshare repository (10.6084/m9.figshare.9708083).
