# Supplementary material for: Double Interferometer Design for Independent Wavefront Manipulation in Spectral Domain Optical Coherence Tomography
Source: Sci Rep. 2019 Oct 10;9:14651. doi: 10.1038/s41598-019-50996-2 (PMC6787192; doi:10.1038/s41598-019-50996-2)
Supplement: Supplementary file 1 — Supplementary Information [file 41598_2019_50996_MOESM1_ESM.pdf]

Double Interferometer Design for Independent Wavefront Manipulation in Spectral Domain Optical Coherence Tomography (Supplementary Material)

Jonas Kanngiesser[1,*], **Maik Rahlves**[1], **and Bernhard Roth**[1,2]

[1]Leibniz Universität Hannover, Hannoversches Zentrum für Optische Technologien, Nienburger Straße 17, Hannover, D-30167, Germany
[2]Cluster of Excellence PhoenixD (Photonics, Optics, and Engineering - Innovation Across Disciplines), Hannover, D-30167, Germany
[*]jonas.kanngiesser@hot.uni-hannover.de

Analytic OCT Signal

The presented OCT design consists of two cascaded interferometers. We start the theoretical discussion by first considering a conventional SD-OCT system which is described within the model of a one dimensional Michelson interferometer[1,2] (right panel of Fig. S1). We assume the field incident to the conventional system to be $E_i'(k,\omega)$. The beam is divided by a beam splitter, reflected at the interferometer reference and sample arm and recombined. In case of an ideal 50:50 beam splitter the intensity at a detector acquiring the power spectral density of the recombined beams reads[1]:

$$i_d(k) \propto \frac{1}{4}\left\langle \left|E_i'(k,\omega)\right|^2 \right\rangle \left| r_r e^{i2kz_r} + a_s(k) \right|^2 \tag{S1}$$

where k denotes the wavenumber, r_r is the reference arm amplitude reflectivity, z_r the reference arm length and the angle brackets denote integration over the detector response time. Propagation through the reference arm imposes the complex phase factor e^{i2kz_r} on the field. The term $a_s(k)$ describes the field amplitude returned from the sample arm.

Taking the interferometer at the source arm into account yields correspondingly:

$$E_i'(k,\omega) = \frac{1}{2}\left(r_1 e^{i2ks_1} + r_2 e^{i2ks_2}\right) E_i(k,\omega) := b(k)E_i(k,\omega) \tag{S2}$$

$s_{1,2}$ describe the respective interferometer arm lengths and $E_i(k,\omega)$ the source field. We consider the amplitude reflectivities of the interferometer arms to be $r_{1,2} = \sqrt{R}e^{i\phi_{1,2}}$, respectively. In this case the one dimensional model can be used to

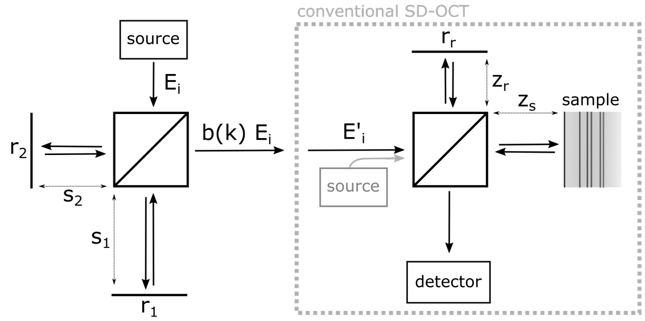

Supplementary Figure S1. Analytical model of the double interferometer OCT design. The model is based on the description of a conventional SD-OCT system in terms of a Michelson interferometer[1,2]. We introduce an additional interferometer at the source of the system, whereas $s_{1,2}$ describe the optical path lengths at the interferometer arms and $r_{1,2}$ the reflectivities.

describe the effect of uniform phase delays $\phi_{1,2}$ which are applied to the two beams reflected at the source interferometer. With the experimental setup this is implemented by the spatial light modulator.

The power spectral density detected at the spectrograph may be written as

$$i_d(k) \propto |b(k)|^2 \cdot \frac{1}{4} \left\langle |E_i(k,\omega)|^2 \right\rangle \left| r_r e^{i2kz_R} + a_s(k) \right|^2 \tag{S3}$$

$$= B(k) \cdot i'_d(k) \tag{S4}$$

with $B(k) = |b(k)|^2$. In the absence of the source interferometer, i.e. in case of $b(k) = 1$, the detected spectral power density reads $i_d(k) = i'_d(k)$, which is the signal expected for a conventional SD-OCT system (compare Eq. S1).

The A-scan signal is acquired from the inverse Fourier transform of the detected power spectral density:

$$I_d(z) = F^{-1}\{B(k)\} \circledast I'_d(z) \tag{S5}$$

where $\circledast$ denotes the convolution operator and $I'_d(z) = F^{-1}\left\{i'_d(k)\right\}$ corresponds to the signal one would receive with the conventional SD-OCT system, i.e. in the absence of the source interferometer. Calculating the inverse Fourier transform of $B(k)$ (Eq. S2) yields

$$F^{-1}\{B(k)\} = \frac{1}{4}\left[2R\,\delta(z) + R\,e^{i\Delta\phi}\,\delta(z+2\Delta s) + R\,e^{-i\Delta\phi}\,\delta(z-2\Delta s)\right] \tag{S6}$$

with $\Delta s = s_1 - s_2$, $\Delta\phi = \phi_1 - \phi_2$ and $\delta(z)$ being Dirac's delta function. The A-scan detected with the double interferometer setup thus reads:

$$I_d(z) = \frac{1}{4}\left[2R\,I'_d(z) + R\,e^{i\Delta\phi}\,I'_d(z+2\Delta s) + R\,e^{-i\Delta\phi}\,I'_d(z-2\Delta s)\right] \tag{S7}$$

Semi-Analytic Simulation

We consider a layered sample consisting of N reflective layers located at different depths. The field reflected from the sample within the one dimensional model yields[1]:

$$a_s(k) = \sum_{n=1}^{N} r_{sn} e^{i2kz_{sn}} \tag{S8}$$

where r_{sn} describes the amplitude reflectivity of the n-th sample layer and z_{sn} is the displacement of the respective layer from the beam splitter.

We implemented a Matlab script to explicitely evaluate Eqs. S1, S2 and S8 at an equally sampled wavenumber grid and with a Gaussian source spectrum. The OCT signal is acquired by calculating the inverse Fast Fourier Transform (IFFT) of the spectral power density (Eq. S1). The resulting A-scan is complex-valued and features Hermitian symmetry.

For the data presented in Fig. 2 we assumed the source spectrum to be centered at 830 nm and chose a rather small bandwidth of 5 nm which results in broad OCT signal peaks (low axial resolution) and better visibility of the data presented. We repeated the calculation for different values of ϕ_1 and fixed ϕ_2. The relative phase shift of the resulting OCT signal is illustrated in Fig. 2.

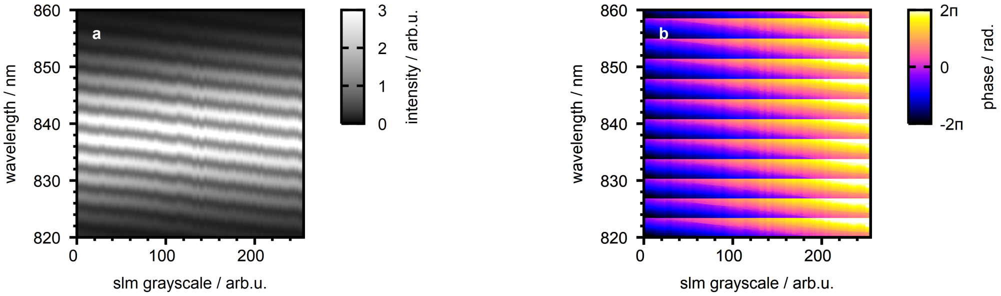

Supplementary Figure S2. Principle of the wavelength dependent SLM characterisation (a) Low-pass filtered spectra acquired with different uniform patterns applied to the SLM for phase shifting. (b) Unwrapped phase of the cosine intensity modulation detected at the individual wavelengths.

SLM Calibration

We implemented a modified calibration procedure based on the work of Fuentes et al.[3]. The procedure uses the SLM to create two beams which interfere at an imaging sensor and applies a constant phase shift to one of the beams. The resulting change in the interference pattern allows to quantify the phase shift.

We illuminate the SLM with a single collimated beam and use the device to create two distinct beams upon reflection (probe and reference beam). In contrast to the previous work, our algorithm uses the SLM to create a circular aperture at the respective beams which results in a clearer diffraction pattern. A CMOS camera is placed at the diffracted beams without further optical elements. By applying a binary grating to the reference beam the first diffraction order is overlapped with the sample beam at the camera and a linear fringe pattern is observed. Changing the grayscale of the SLM pattern at the probe beam applies an unknown phase shift which, in turn, results in a shift of interference fringes. For the calibration procedure we capture cross-sectional intensity profiles of the linear fringe patterns observed for different grayscales applied to the sample beam. One-dimensional fringe tracking allows to determine the phase shift corresponding to the grayscale value applied to the SLM screen.

We investigated the phase shifting characteristics of the SLM with the broadband SLD source which is also used for OCT signal acquisition. The procedure is expected to work with this kind of source in case the coherence length is larger than the path length difference between the reference and the probe beam. The latter value can be modified by changing the beam positions at the SLM and the distance between the SLM and the imaging sensor. After characterisation we modified the gamma look-up-table of the SLM to provide linear 2π phase modulation with the SLD source[3].

SLM Dispersion

We determine the dispersion of the SLM by imaging a single reflecting sample with the SD-OCT system while using the SLM to manipulate the phase difference $\Delta\phi$ between reference and sample beam (compare Fig. 3). In this case, the detected spectral power density reads[1,4]:

$$i_D(k) = A(k) + B(k) cos(2k(z_r - z_s) + \Delta\phi) \tag{S9}$$

We consider the phase shift $\Delta\phi$ to be wavelength dependent and the parameters $A(k)$ and $B(k)$ to depend on wavelength only. With a single reflecting sample, phase modulation by the SLM results in a cosine intensity modulation at each wavelength, i.e. at each individual pixel of the spectrograph. The wavelength dependent phase shift can, thus, be estimated from the phase of the intensity oscillation.

We investigate the dispersion of the SLM with a protected silver mirror taken as sample and acquire a set of spectra while using the SLM for uniform phase manipulation at the reference beam which corresponds to manipulating $\Delta\phi$. To account for OCT imaging artefacts, we calculate the A-scans from the detected spectra and low-pass filter the signals such that only the DC and the mutual interference components are kept. The signal is then back-transformed to the spectral domain (Fig. S2a). The phase shifting characteristics of the SLM is found from the inverse cosine of the intensity modulation detected at each pixel of the spectrograph and subsequent phase unwrapping (Fig. S2b). We alternate the acquisition of the phase shifted spectra with reference acquisitions which correspond to the signal taken with zero phase shift applied. Subtracting the phase of the reference signal corrects for temporal phase drift during the acquisition as well as for the phase offset which is observed in Fig. S2b.

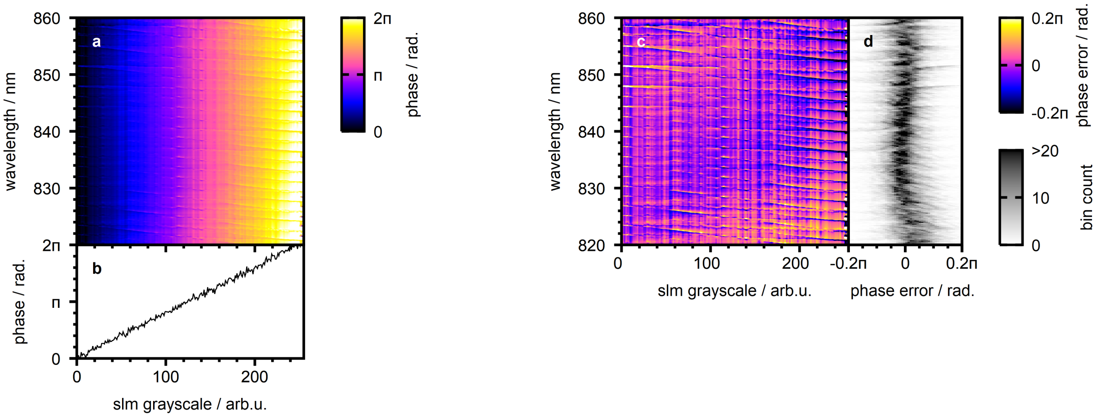

Supplementary Figure S3. Dispersion of the SLM. (a) Spectrally resolved phase shift of the SLM. The data is acquired by subtracting the phase of the reference spectra from the data displayed in Fig. S2b. (b) Spectral average of the observed phase shift. (c) Phase difference to the polynomial fit of the average phase shift. (d) Histogram of the phase differences. Each row in d illustrates a color-coded histogram of the corresponding row in c.

The resulting wavelength dependent phase modulation characteristics of the SLM is illustrated in Fig. S3a. Panel b features the wavelength averaged phase modulation which is observed to agree well with the linear 2π modulation for which the SLM was calibrated. To illustrate the wavelength dependency of the observed phase shifting characteristics more clearly, Fig. S3c depicts the phase error which we consider to be the difference of the applied phase shift and the polynomial fit to the wavelength averaged data. Large phase errors are observed near those positions where extrema of the cosine intensity modulation are found with the original spectral data. Near the extrema small intensity fluctuations result in large phase errors and phase jumps which we consider to be measurement artefacts. Fig. S3d illustrates the color-coded histograms of the phase error observed at each wavelength. The figure gives an idea of the overall wavelength dependency of the applied phase shift. Based on the data we find the phase shift applied by the SLM to be accurate within $\pm 0.1\pi$ for the spectral range considered. We assume this value to be negligible for the experiments presented.

Spectral resolution of the OCT system

The average wavenumber sampling of the spectrograph was found to be $\Delta k = 3.61$ cm^{-1} resulting in a maximum single-pass imaging depth of $z_{max} = 4.4$ mm. We assume a pixel fill factor near unity and, thus, the spectral range δk_{pix} covered by each pixel to match the spectral sampling Δk. The resulting axial signal intensity drop is expected to correspond to the inverse Fourier transform of a rectangular function[2] which is proportional to $sinc(\delta k_{pix} z/2)$ with respect to the double-pass path length scale z. We further estimated the optical resolution of the spectrometer by taking the OCT signal from a strongly reflecting sample and investigating the axial signal drop while changing the reference arm length. We corrected the signal for the sensitivity loss expected from the finite pixel size and calculated a Gaussian fit. The sensitivity drop resulting from the optical resolution is expected to be proportional to $exp[-\delta k_{opt}^2 z^2/(16\,ln(2))]$ if a Gaussian point spread function (PSF) is assumed[1]. The fit parameter δk_{opt} describes the FWHM width of the PSF and is found to be 6.58 cm^{-1}. To illustrate the cumulative effect of the finite spectral resolution on the expected OCT signal the product of both functions is drawn to Fig. 2.

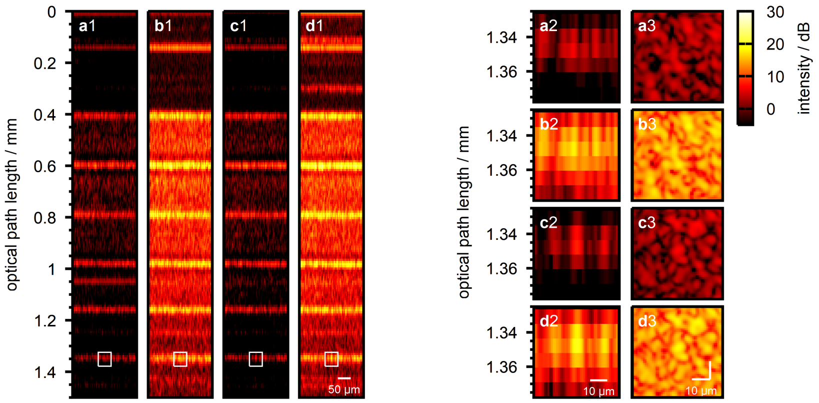

Supplementary Figure S4. Lateral sample scan with wavefronts shaped by the adaptive algorithm. (a1) B-scan taken with flat wavefront applied to both beams. (a2) B-scan close-up centered at the target scan position which was chosen for wavefront optimization. (a3) En-Face scan at the target depth ($z = 1.35$ mm) for wavefront optimization. (b1-b3) Same as a1-a3 with adaptive wavefront correction at the reference beam (Fig. 4e). (c1-c3) Same as a1-a3 with adaptive wavefront correction at the sample beam (Fig. 4d). (d1-d3) Same as a1-a3 with both optimized wavefronts applied to the respective beams simultaneously.

Lateral sample scan with adaptive optics correction

We repeated the experiment presented in Fig. 6 with the phase patterns found by the sensorless adaptive algorithm (Fig. 4). Fig. S4, panels a1-a3 illustrate the B-scan and En-Face scan taken with a flat wavefront applied to the sample and to the reference beam. Fig. S4, panels b1-b3 illustrates the same data in case the wavefront is optimized at the reference beam (Fig. 4e). As with the iterative wavefront correction at the reference beam, we observe the overall signal to be enhanced. The correlation coefficient of the two En-Face scans (Fig. S4 panels a3 and b3) reads 0.90. As compared to the iterative correction, the application of the smooth wavefront found by the adaptive algorithm results in a larger signal enhancement (compare Fig. 6 panels b1-b3 and Fig. S4 panels b1-b3). In case the sample is scanned with the wavefront optimized at the sample beam (Fig. 4d) we observed no significant signal enhancement at the given intensity scale (Fig. S4 panels c1-c3). The correlation coefficient with the En-Face scan taken with flat wavefront (Fig. S4 panels a3 and c3) reads 0.13. When applying both optimized wavefronts to the respective beams simultaneously the overall signal is found to be enhanced as compared to the acquisition with the flat wavefront. The signal enhancement is dominated by the effect of wavefront shaping at the reference beam. We further observe the image to be correlated with the scans taken with wavefront optimization at the sample beam alone. The correlation coefficient of the En-Face scans (Fig. S4 panels c3 and d3) is found to be 0.89.

References

1. Drexler, W. & Fujimoto, J. G. *Optical coherence tomography: technology and applications* (Springer Science & Business Media, 2008).

2. Leitgeb, R. A., Hitzenberger, C. K. & Fercher, A. F. Performance of fourier domain vs. time domain optical coherence tomography. *Opt. Express* **11**, 889–894 (2003).

3. Fuentes, J. L. M., Fernández, E. J., Prieto, P. M. & Artal, P. Interferometric method for phase calibration in liquid crystal spatial light modulators using a self-generated diffraction-grating. *Opt. Express* **24**, 14159–14171 (2016).

4. Targowski, P. *et al.* Complex spectral oct in human eye imaging in vivo. *Opt. Commun.* **229**, 79–84 (2004).
